# Human Stem Cell-Derived Extracellular Vesicles: A Pioneering Path from Biogenesis to Cerebral Ischemic Stroke Therapy

**DOI:** 10.3390/ijms262110550

**Published:** 2025-10-30

**Authors:** Anamika Roy, Tristan Driscoll, Samuel C. Grant, Yan Li

**Affiliations:** 1 Department of Chemical and Biomedical Engineering, FAMU-FSU College of Engineering, Florida State University, Tallahassee, FL 32310, USA; ar21cf@fsu.edu (A.R.); tdriscoll2@fsu.edu (T.D.); 2 National High Magnetic Field Laboratory, Florida State University, Tallahassee, FL 32310, USA

**Keywords:** stroke, extracellular vesicles, human stem cells, biogenesis

## Abstract

Despite advances in modern medicine and increased public awareness, cerebral stroke remains a leading cause of death and long-term disability worldwide. With over 600,000 new cases annually, innovative therapeutic strategies are being explored to enhance recovery outcomes. One promising approach is the use of human stem cell-derived extracellular vesicles (EVs), particularly exosomes, which function as mediators of intercellular communication. EVs have emerged as pivotal mediators of intercellular communication with immense potential in therapeutic applications. This review discusses the pioneering journey of EVs from their biogenesis and molecular cargo loading to their translation into clinical strategies for cerebral ischemic stroke therapy. While direct stem cell transplantation has faced limitations such as immune rejection, tumorigenicity, and short shelf life, human stem cell-derived EVs offer a cell-free alternative with enhanced safety, stability, and functional versatility. Preclinical studies reveal their capacity to modulate inflammation, protect neural tissue, and promote recovery through the transfer of bioactive molecules. Additionally, EVs isolated from biofluids such as blood and cerebrospinal fluid serve as promising biomarkers for stroke severity and prognosis. Despite this promise, several challenges persist—from standardizing isolation techniques and optimizing therapeutic cargo to scaling up production for clinical-grade use. This review critically examines the current understanding of EV biology, highlights the advances in stroke-related applications, and outlines key hurdles that must be addressed to unlock their full therapeutic potential.

## 1. Introduction

Strokes, predominantly of the ischemic variety, are one of the most common causes of death and disabilities in the world. In the United States alone, one person suffers a stroke every 20 s and dies due to stroke every 3 min and 14 s [1,2]. Moreover, during the pandemic, even with the availability of world-class treatment, 4.73% of the population of the US died of stroke, and the stroke rate increased by 26.3 in 2024 [1,2]. An ischemic stroke occurs when blood flow in a specific part of the brain is restricted, usually by a blood clot. The frontline treatments of this condition include thrombolytic drugs (i.e., tissue plasminogen activator, TPA) and surgical thrombectomy (removal of blood clot), followed by rehabilitation with or without physical therapy. These treatments can only be applied over a 4.5 h window, which is extended up to 24 h in some instances for surgical intervention and depending on the severity of the condition [3,4]. After a stroke, most patients suffer depression, physical difficulty, or some form of disability, necessitating at least physical rehabilitation.

For treatment beyond clot removal, clinical stem cell therapy has shown promise. Preclinical and clinical studies have demonstrated that stem cell therapy can provide neuroprotective, anti-inflammatory, angiogenic, and neurogenic effects [5,6]. Due to some limitations of the therapy (e.g., low storability of stem cells), extracellular vesicles (EVs) or the small-sized EV subpopulation exosomes (40–200 nm) derived from human stem cells have recently been attracting a lot of attention from researchers for stroke treatment [7]. EVs are lipoprotein-membraned vesicles secreted by cells as a means of communication and maintenance. When EVs were discovered, they were identified as micro- or nano-vesicles containing cellular waste. However, current research indicates that a variety of EVs perform different functions, with the exosome class comprising a unique subtype [8]. Overall, EV subtypes, including micro-vesicles (50 to 1000 nm) [9], apoptotic bodies (>100 nm), and exosomes (40 to 200 nm), are classified by cellular origin, biogenesis, size, content, and function [10], as well as surface protein markers [11]—all of which can be impacted by the purification and separation methods employed [12]_._ EVs can be secreted by all cell types and found in several biofluids in mammals [8], generally containing nucleic acid (e.g., mRNA and microRNA), proteins, and lipids, depending on their source [13,14,15]. According to their intended therapeutic or diagnostic use, EVs are derived or collected from a specific source (like biofluids) or cultured cells. For example, exosomes derived from stem cells have growth factors and anti-inflammatory properties, and they can improve functional and behavioral recovery [16]. They can also be used as potent biomarkers of different diseases, including cancer [17,18]. Their nanometer size makes EVs viable for penetrating biological barriers in the body. So, EVs are considered as a viable alternative for directly implanted stem cell-based therapies (e.g., for drug delivery purposes), while endogenous EVs may provide diagnostic capabilities.

For exosomes, identifying the contents that are responsible for therapeutic effects is critical. In some investigations, the microRNA (miRNA) found in exosomes potentially contributes to the recovery of the ischemic penumbral region [19]. There are also some identified proteins that are responsible for different therapeutic properties, such as angiogenesis and anti-inflammatory immune modulation. In addition to cell-derived exosomes, bioengineered exosomes can be utilized for drug delivery [20]. Specifically for neural diseases, the size of exosomes makes them a suitable candidate for drug delivery systems to the brain [21], as they can cross the blood–brain barrier (BBB) readily [22]. Moreover, some exosomes derived from stem cells have also shown neuro-regeneration effects [23]. This work reviews challenges involving EV therapeutics in light of the current state of EV research, from biogenesis to clinical application of stem-cell-derived EV therapy for ischemic stroke. The goal is to highlight the challenges relating to this field of research for new researchers.

## 2. EV Biogenesis and Bioengineering

**Figure 1:**
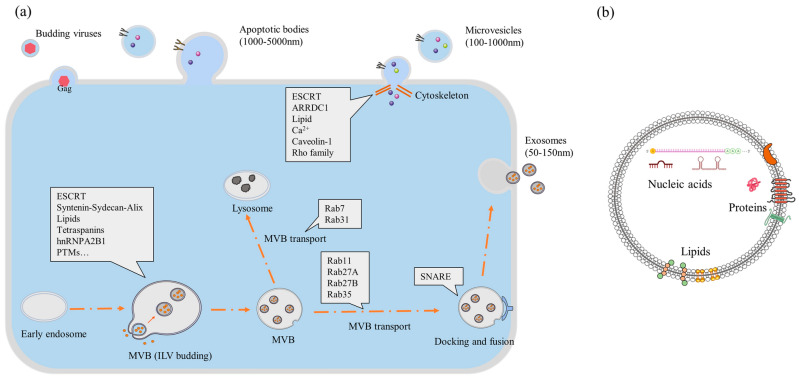
Biogenesis of EVs from cells (generic): (**a**) the biogenesis route of three different kinds of EVs and involved proteins in the cell; (**b**) contents of an EV within its lipid bilayer [37] [abbreviations—ESCRT, endosomal sorting complex required for transport; MVB, multivesicular body; ARRDC, arrestin domain-containing protein].

There are several pathways for biogenesis depending on the type of EVs (Figure 1), which are important as EVs can be used as a direct therapy, a diagnostic medium, or a medium for drug delivery [24]. Their external membrane of lipoprotein and size make EVs one of the most biocompatible drug delivery systems. There are three well-established biogenesis mechanisms for different types of EVs.

Most of EV biogenesis in cells occurs along the endo-lysosomal pathway [25]. In simple terms, inward budding or surface blebbing initiates the process within cell organelles, such as lysosomes, retrosomes, and Golgi bodies [26]. Sorting of proteins is mostly managed and transported by a family of proteins associated with a pathway for the endosomal sorting complex required for transport (ESCRT) [27]. When transported to the inner side of the plasma membrane, proteins for encapsulation are anchored by SNARE proteins, after which EV budding occurs. Primarily, the budding is facilitated by the redistribution of the plasma membrane. Multivesicular bodies (MVBs) are generated and can be released directly from the cell. If MVBs are not released, then a second biogenesis mechanism can take place that results in smaller EVs called exosomes. In this biogenesis scenario, intraluminal vesicles (ILVs) are formed inside the intracellular MVB. ILVs are later released as exosomes after reabsorbing the lipoprotein membrane of MVBs [28,29]. The process is controlled by ESCRT, cellular lipid, ionic calcium, and other proteins (mostly of the Rho and Rab families) [27]. It is still not clear why different sizes of EVs are released and what mechanisms impact size differences, but the most plausible reason is their cargo size. MVBs are transported to the plasma layer, and the union of MVBs with the plasma membrane is responsible for the bulk release of exosomes [26]. Many enzymes, cellular proteins, and other mechanisms are involved in the process [28,30]. The biogenesis of MVBs and exosomes is still being researched extensively to determine which factors most affect the process.

On the other hand, apoptotic body biogenesis is more straightforward, as it results directly from cell apoptosis. Apoptotic bodies are the largest among EVs, but they are also the most undefined. Their size can vary based on the cargo, and, in some cases, they can even carry whole cell organelles [31]. Though mechanisms of EV biogenesis are specific, the process could have different sets of genes and proteins based on specific cell types, the condition of the donor of cells, culture conditioning, and function [32,33,34]. Therefore, the source is an important concern in the bioengineering process [35,36].

For diagnostic purposes, EVs (apoptotic bodies or exosomes) are extracted from biofluids or cell lysates. For their therapeutic potential, EVs are harvested from viable cells. However, apoptotic bodies are not considered to have therapeutic potential and are not usually evaluated. Deriving EVs from cells is difficult due to their sensitive nature and because the influence of culture conditions on biogenesis is not well understood [38]. Currently, there are some guidelines for bioengineering EVs. First, the source cells must be in a suitable condition to trigger secretion of the desired kind of EVs. To have therapeutic effects, the desired EVs should not face diffusional constraints in vivo. For example, they must not adversely interact with physiological barriers, such as blood vessel epithelium. Second, EVs should be engineered to target cell types preferentially relevant to specific disease pathogenesis, e.g., angiogenesis and prevention of neural cell death in an ischemic stroke region. The separation process can also impact the quality of EVs. Finally, research is needed to optimize the mass production of effective EVs efficiently.

Engineering of EVs can be performed by two methods. One approach is the passive exposure of EV-producing cells to supplied genetic materials during conventional cell culture. Another approach is a more direct EV modification. Both methods require EVs to be harvested from cells. Using different bioreactors and cell conditioning techniques, engineers are trying to produce a high yield of cells of a desired quality for EVs. Specific preconditioning of mesenchymal stem cells (MSCs) has been shown to improve and target desired therapeutic effects [39]. Preconditioning affects the secretion properties of the cells. To maximize cell proliferation, 3D cultures or bioreactors can be used. The type of bioreactor can directly affect the secretion properties of cells, especially for scaled-up production [40]. Recently, vertical wheel bioreactors have been used for large-scale stem cell culture [41,42]. Due to the unique hydrodynamic characteristics of the reactor, stem cell production is enhanced [43]. Researchers have also proven that using vertical wheel bioreactors for cell culture can increase EV production while also having a positive influence on EV content [44].

After culture, cells are separated, and then EVs are isolated and purified from either cell media or cell lysates using a series of centrifugation steps. Even after isolation, EVs can be modified for their intended use by direct EV modification [45]. Depending on testing conditions, EVs can be marked with magnetic or bio-luminescence markers. In many neurodegenerative investigations using EV therapy in animals, EVs have poor target specificity. Most of the injected EVs via intravenous routes have been cleared within a few days via the spleen, kidney, and liver [46,47]. But recently, there have been developments in more specifically loading and targeting EVs, with target specificity improved by incorporation of a binding protein [48,49]. This kind of bioengineering opens new doors for drug delivery methods.

## 3. EV Separation and Purification

EVs are either collected from biofluids or cell culture media. In both cases, the fluid containing these EVs has some impurities, such as cells or cell debris, soluble or insoluble proteins or lipoproteins, occasionally some fractioned genetic material, or pathogens. Purification may affect the contents and size distribution of EVs [50]. So, EV purification is not only highly important for further analysis but also necessary for further EV classification processes.

The most common method used for EV isolation is ultracentrifugation [51]. Initially, EVs were purified from cell culture media using a differential ultracentrifugation method [8]. It ranged from 80,000× *g* to 100,000× *g* centrifugation speeds [8]. Now, high centrifugation speeds of up to 140,000× *g* are performed routinely to separate EVs based on size [52]. The standardization of EV separation and characterization protocols was established by the International Society for Extracellular Vesicles (ISEV), through guidelines established in a position paper on the minimal information for studies of extracellular vesicles (MISEV) [53,54]. Updated every four years, the guidelines establish best practices for three specific research conditions based on the intended purpose of the EVs: EV discovery and characteristics research, diagnostic EV research, and preparatory EV research for therapeutic purposes [55]. Sample collection and isolation procedures will vary for each of these conditions. EV discovery and characteristics research aims to find target characteristics in specific kinds of EVs, usually based on size. Diagnostic EV research is focused on identifying biomarkers correlating to specific diseases. Preparatory EV research is usually for preclinical or clinical trials and is more strictly regulated.

For EV discovery and characteristics, EVs are isolated from cell culture media using differential ultracentrifugation [54]. For diagnostic purposes, EV isolation is complicated by the biofluid source. Blood serum and cerebrospinal fluid (CSF) are the most used biofluids for ischemic diagnostic purposes [56]. After purifying from cell debris, these biofluids are diluted usually with PBS [55]. Then, EVs are isolated using either ultracentrifugation, gravity-dependent size occlusion, immunoaffinity isolation, polymeric precipitation, or microfluidics techniques [55]. Density-based isolation methods, such as centrifugation and ultracentrifugation, are the most common, efficient, and least likely to damage EVs [57]. A series of centrifugation and ultracentrifugation is performed to purify the desired EVs. But, these kinds of density-based separations are considered more as an enrichment technique of EVs rather than true isolation [57]. Ultracentrifugation can be a quite expensive, time-consuming, and labor-intensive process. Gravity-dependent size occlusion, such as ultrafiltration or gel filtration, can be used for specific types of EVs combined with ultracentrifugation [58]. Though the process is quite simple and quick, there could be deformation or breakage issues for larger EVs [59]. On the other hand, immunoaffinity isolation is used to isolate EVs based on specific protein markers (such as CD45). So, it is highly antigen-specific compared to other techniques [60]. It is quite useful in diagnostic purposes for isolating EVs from serum or plasma samples [61,62]. Polymeric precipitation is especially used for exosome isolation from biofluids. It is a process of isolating EVs using a precipitating polymer (e.g., polyethylene glycol (PEG) or mannuronate–guluronate polymer (MGP), sodium acetate) [59]. Precipitation is not a very EV-specific form of separation. Other separation or purification steps might involve using microfluid devices or kits. One method involves an antibody-coated microfluid device in filtered cultured media and or biofluid to separate smaller EVs [63].

## 4. EV Classification

As aforementioned, there are three categories of EVs depending on size and biogenesis. But in similar types of EVs, contents can vary widely depending on the cell source, culture conditions experienced by that cell source, EV extraction approaches, and purification methods [53,54,64]. Among all EVs, exosomes are the most well studied for therapeutic applications. In most instances, EVs are collected using xeno-free and EV-depleted culture media of mammalian cells at the expense of lower cell proliferation [65] and lower EV production [54,66]. Some EVs derived from biofluids have components that can be used as a diagnostic biomarker, and some exosomes can be helpful in treating disease [67]. Thus, the contents of EVs are an important attribute for their functionality. Proteomics, genomics, and microRNA sequencing studies are used to analyze various proteins and nucleic acids, such as fragments of DNA and miRNA, which are found in EVs [68,69,70]. Initially, cargo identification was pursued, but, more critically, the role that EV content plays under the conditions being investigated must be determined. Furthermore, the relative proportion of proteins and nucleic acids within individual EVs and across EV populations is impacted by culture conditions, cell source, and cell passage [71].

ISEV has set definitions for EV classification as well as expected contents. For characterization purposes, EV samples from a specific source and purification procedure must undergo global quantification, potentially measuring size, total protein amount, or total lipid amount, preferably relative to the number of particles or cells employed as a means of measuring purity and measurement reliability [54]. Besides the source and isolation method, general characterization as an EV mandates the evaluation of at least one expected protein from each of the following three classes: transmembrane or lipid-bound proteins; cytosolic proteins; and/or intracellular proteins [53]. Transmembrane proteins are generally found on the EV surface. Examples of these kinds of proteins are tetraspanins (such as CD4, CD9, CD45, CD63, CD81, CD82, CD86, MHC-I, and MHC-II), integrins, and lactadherin. They are very important in engineering EV (especially exosome) biogenesis, and predicting functionality [72]. Sometimes, transmembrane proteins can be indicative of the source of the EV. For example, CD63, CD81, or CD9 are found in EVs derived from MSCs or their derivatives [11,73]. Cytosolic proteins are enzymes found inside EVs, which have membrane or receptor binding properties as well as protein synthesis properties. Heat shock proteins (e.g., HSP70) and microtubule-associated proteins (e.g., Tau) are examples of these kinds of proteins. If found on the EV membrane, they may increase target specificity [74]. Unlike these two protein groups, intracellular proteins are not commonly found in exosomes but in other types of EVs (such as apoptotic bodies). These proteins are mostly components of cellular organelles [53]. Examples include histones and cytochromes. The proteins associated with non-EV co-isolated structures represent those materials that may be shed or secreted by cells or during cell culture but not encapsulated in an EV, and they can be used as a purity control [53]. These materials include lipoproteins, albumin, ribosomal proteins, and nucleic acid aggregates, and they should be absent from pure EV preparation. Global proteomics analysis, as well as the identification of the desired protein using Western blot, ELISA, the specific protein assay, or high-resolution flow cytometry, is a must for EV classification.

ISEV also sets standards for single EV quantification to assess the EV size range. This analysis should consist of one electron microscopy (EM) and one non-EM measurement. For this purpose, some advanced techniques, such as nanoparticle tracking analysis (NTA), dynamic light scattering (DLS), Ramen spectroscopy, and Scanning EM, can be used. Though imperfect, this quantification provides an estimation of the number of particles per volume and the particle size distribution [75]. The data can be interpreted with respect to general characterization and cargo determination. Although not required by the ISEV recommended classification, there is a strong suggestion that the topology of EVs also be characterized with respect to the anticipated active components, through means of digestion, permeabilization, and possible modification for drug delivery and target specificity [76,77,78]. For all research purposes, these measurements are performed prior to use. However, as research progresses, new metrics could be added to the updated guidelines for the ISEV classification standard.

## 5. Nucleic Acid Cargo in EVs Relevant to Ischemia

EVs are rich in many nucleic acids, such as microRNA (miRNA), messenger RNA (mRNA), long non-coding RNA (lncRNA), circular RNA (circRNA), small interfering RNA (siRNA), surface DNA, and mitochondrial DNA (mtDNA) [79,80,81]. Due to size restrictions, EVs generally do not contain unfragmented long RNA or DNA. These nucleic acids, though not fully explored, can be used as biomarkers as well as therapeutic cargo (Table 1).

In some studies, certain RNA strains were found to be upregulated in EVs collected from the biofluids of patients. Different types of exosomal miRNAs are found up- or downregulated in the blood serum of patients at different times during the progression of or recovery from stroke. In acute ischemic stroke, patients’ blood serum exosomes had upregulated miRNA-134 within 24 h [82]. In the subacute phase of stroke, miRNA-422a and miRNA-125b-2-3p of human blood serum exosomes were decreased compared to the acute phase, and miRNA-422a was increased in the acute phase [83]. These two miRNAs could act as biomarkers of the temporal evolution of ischemic stroke, as their expression in serum exosomes also decreased with functional improvement following stroke. Likewise, differential stroke-related expression was identified for miRNA-9 and miRNA-124 found in blood serum exosomes, as well as exosomal miRNA-21-5p and miRNA-30a-5p [84,85]. These miRNAs were found to be upregulated as stroke injury was aggravated, permitting them to be used as biomarkers of stroke severity. During an in vitro experiment of primary hippocampal neuronal cells of stroke mice, miRNA-PC-5P-12969 was upregulated and secreted in exosomes after 4 h [86]. The study also found normal levels of this miRNA in unaffected parts of the brain and during the post-stroke recovery stage. So, the authors concluded that miRNA might be upregulated to improve homeostatic imbalances experienced during stroke.

EVs may provide markers for transient ischemic events, which can be difficult to capture with imaging studies. miRNA-125a-5p, miRNA-125b-5p, and miRNA-143-3p in blood samples are elevated during transient ischemia, with further upregulation during acute ischemic stroke [86,87]. Circular RNA derived from oxoglutarate dehydrogenase (CircOGDH) can be found enriched in neuron-derived exosomes from the ischemic penumbra region of mice [88]. A human study found that miRNA-126 has angiogenic properties and contributes to a temporary improvement in homeostasis following stroke [89]. Thus, upregulated miRNA-126, which is found in stem cell-derived exosomes, has the potential to reduce the risk of stroke as well as improve homeostasis of the penumbra region. In animal models, the overexpression of miR-124 in brain injury has been found to promote neuronal differentiation [90]. miR-124 has some degree of neurogenesis effect in the adult brain. In another study, miRNA-21-5p and miRNA-30a-5p were found to be upregulated in acute ischemia but downregulated during recovery [87]. Another study of acute ischemic patients found that upregulated expression of miRNA-223 in exosomes in blood serum indicates a poor recovery rate for patients [91]. It has pro-inflammatory effects that foster further neurodegeneration in stroke patients [92]. Additionally, other circulating long non-coding RNAs that are not necessarily contained in EVs can regulate the aforementioned miRNAs to have significant impacts on ischemic recovery [93]. In vitro analysis related to exosomal RNAs has been shown to promote neurogenesis applicable in ischemic stroke. For example, miRNA-21a was found in EVs secreted by neural progenitor cells (NPCs), which enhances neurogenesis [94].

**Table 1:** RNA expressed in EVs isolated from serum and neurons as biomarkers and potential therapeutic agents in ischemic stroke.

miRNA/RNA	Expected Activities	Ref.
miRNA-134	Potentially a novel biomarker for diagnosis and prognosis.	[82]
miRNA-422a and miRNA-125b-2-3p	Potential biomarker for prognosis of the acuteness of stroke.	[83]
miRNA-9	Biomarker for assessing the extent of injury caused by ischemic injury.	[84]
miRNA-21-5p and miRNA-30a-5p	Biomarkers for acute and hyperacute ischemic stroke.	[85]
miRNA-PC-5P-12969	Potential candidate for ischemic stroke peripheral marker but also a drug target for ischemic stroke.	[86]
miRNA-125a-5p, miRNA-125b-5p, and miRNA-143-3p	Potential biomarkers for transient and acute ischemic stroke.	[87]
CircOGDH expression	Upregulated expression in the penumbra tissue of ischemic mice.	[88]
miRNA-126	Regulators of angiogenesis and endothelial cell function, found in stem cell-derived exosomes.	[89]
miRNA-124	In animal models, promotes neurogenesis. In humans, can be used as a biomarker of ischemic injury.	[84,90]
miRNA-21-5p and miRNA-30a-5p	Can be indicative of the recovery phase of ischemia in human patients.	[85]
miRNA-223	Upregulation in blood serum indicates poor recovery of patients.	[91]
miRNA-21a	Enhance microenvironment necessary to promote neurogenesis of induced NPC in vitro.	[94]

[Abbreviations—RNA, ribonucleic acid; miRNA, microRNA; NPC, neural progenitor cell].

## 6. In Vivo Detection and Tracking of EVs

The specificity and mechanism of action used by EVs to target ischemic lesions are still unknown. These processes are critical to evaluate with respect to the use of EVs as a therapeutic agent. To determine target specificity in preclinical and clinical studies, EVs are labeled frequently. There are hardly any endogenous components in EVs that can be used to track them in vivo. As demonstrated by in vitro studies, EV membranes can incorporate features such as transmembrane proteins, transporters, and antigens reflecting their cell origin [95,96]. So, labeling agents should have properties either to fuse these proteins to their membrane or to penetrate inside the lipoprotein membrane (examples are shown in Table 2).

Most detection modalities require a labeling agent to be incorporated with the EVs. This incorporation can use either direct or indirect approaches to label EVs, with most studies focusing on exosomal populations [97]. Direct labeling introduces the labeling agent after EVs are isolated, while indirect approaches label the cell source, which transfers the agent to EVs during biogenesis. Though direct labeling gives the most contrast, the indirect labeling method allows for evaluation of an agent’s potential cytotoxicity based on impacts on the source cells. Indirect labeling gives a subtle contrast in most cases. Most of the indirect labeling agents have been reported as membrane bound. Ideally, a labeling agent should be beneficial or benign biologically to both the cell source and the eventual therapeutic target organ. Furthermore, the interaction and localization of the labeling agent with EVs will impact detectability. Labels can be incorporated internally or through association with the membrane in EV preparations. For imaging-based detection methods, these labeling agents are called contrast agents. For in vivo detection, molecular imaging techniques, such as fluorescence imaging, computed tomography (CT), magnetic resonance imaging (MRI), and positron emission tomography (PET), are most commonly employed [98]. For fluorescent imaging, membrane-associated agents are generally preferred due to accessibility [99]. The ideal labeling agent would be inexpensive, nontoxic, easy to use, as well as sensitive enough to provide a high signal-to-noise ratio.

To observe in vitro cellular uptake of EVs via fluorescent imaging, lipophilic fluorescent dyes are commonly used. There are lipophilic dyes of different strengths and colors. For example, carbocyanine dyes (e.g., DiR, color green) are weakly fluorescent. So, they are used to label mouse MSC-derived exosomes for near-infrared fluorescent imaging [100]. PKH dyes are highly fluorescent and are used both in cell membrane and EV staining. They irreversibly bind to EV membranes, providing green (PKH67) or red (PKH26) fluorescence [101]. But these dyes are reported to aggregate and increase vesicle size [102]. Other lipophilic dyes, such as amine-reactive, thiol-reactive, and azide-specific dyes, have also been used to label EVs. These dyes are quite hydrophobic and difficult for some applications using aqueous buffer [101]. But there are several improved dyes, named Mem, which are stable in aqueous buffers and do not affect the size of EVs [102]. These can be used in direct labeling of EVs with vivid fluorescent colors (green, red, or deep-red) [102]. Compared to PKH, Mem dyes are larger molecules, and the in vivo application of Mem has not been well tested. Although tracking EVs non-invasively via fluorescent imaging is quite challenging, some of these agents have potential for in vivo tracking of EVs via bioluminescent imaging.

For neurological studies of preclinical models, MRI is an excellent modality with both ease and flexibility in incorporating labeling agents. MRI-detectable labels are usually metal-based nanoparticles with specific magnetic properties that induce altered relaxation of surrounding or exchanging water protons. Common MRI contrast agents include gadolinium chelates, iron oxides, and manganese ions. Gadolinium chelates are FDA-approved for clinical use, but have demonstrated mild neurotoxicity [103] and accumulation in the human brain [104] as well as the more widely known issues in some compromised patients with nephrotoxicity [105]. As most of the disadvantages are reported for long-term accumulation, gadolinium is still in use to label exosomes for preclinical studies [106]. Manganese-based agents are also neurotoxic and limited only to investigational studies [107]. On the other hand, superparamagnetic iron oxide nanoparticles (SPION) or ultra-small superparamagnetic iron oxide nanoparticles (USPION) are used for MRI because they have good contrast, are not cytotoxic at low doses, and can be coated or functionalized with proteins [108,109]. Most commonly, SPIO particles are coated with dextran or carboxy-dextran [110], but they can be used in aqueous suspensions too. There are quite a few preclinical studies where SPION or USPION are used to label EVs for tracking cargo delivery to the targeted organ [111,112,113]. In some preclinical studies, iron oxide labeled EVs were used to direct them to the targeted site using magnetic forces [114,115]. Venofer is such a nanosized protein fusion iron oxide particle, which is used to label EVs and proposed for treating tumors using magnetic hyperthermia [115], but it has not been preclinically used yet. On the other hand, ferumoxytol and ferucarbotran nanoparticles are FDA-approved and can be metabolized by humans with minimal side effects. These kinds of contrast agents have an iron oxide core and an organic coating. Based on the coating, the particles can be used as internalized or membrane-associated labels for EVs. As well as impacting surface properties, the coating can increase contrast, solubility, and cellular or EV uptake efficiency [116]. Some researchers use special kinds of proteins for better labeling of EVs and to lower the cytotoxicity of iron oxides. For example, 4-6 nm USPION can label EVs indirectly through biogenesis to provide MRI contrast [112]. Alternatively, a biogenesis route of EV labeling has been achieved through viral transfection of MSCs with the fusion of heavy chain ferritin (FTH1) bound to a truncated lactadherin. In this fashion, transfected MSCs generated EVs in which the fused lactadherin was incorporated into the membrane with normal outer surface localization, providing MRI visibility in vitro and in vivo through the associated iron-containing FTH1 [117]. Gaussia luciferase–lactadherin (fluorescence labeling agent used in bioluminescent imaging) is also a similar fusion protein, which can be used to label EVs by transfecting the secreting cells [118].

Some researchers, especially those studying tumor animal models, have also labeled and detected EVs using complexes with radioactive elements or radioisotopes, such as ^99^Tc-tricarbonyl and ^99^Tc-hexamethylpropyleneamineoxime (HMPAO) [119,120]. They tested nanovesicles mimicking EVs with the labeling agents [119]. Another chelating complex agent using ^111^In can be used to label EVs directly derived from melanoma cells [121]. Most of the labeling agents are radioactive for SPECT and PET applications. CT is also a common in vivo imaging technique in ischemia, heart disease, and cancer. Researchers have tracked EVs with gold nanoparticles (5 nm size) in the brain injected intravenously in mice to assess biodistribution by means of CT [122,123]. Other CT agents, namely, iodine-containing iohexol, can be used to label EVs by passive diffusion or sonication, allowing EVs to provide target homing and a potential platform for drug delivery [124].

**Table 2:** EV markers and labeling agents for in vivo applications.

Labeling Agent	EV Derived From	Detection Method or Use	Reference
DiR	Bone marrow-derived	Used to observe therapeutic effects of exosomes for ischemic stroke by molecular imaging in mice.	[101]
Gadolinium Chelates	Umbilical cord-derived MSC	Used to capture tumor-homing ability of MSC exosome in mice via MRI.	[106]
USPION	USPION-labeled Adipose tissue derived MSC	Used to tract exosomes in the muscle of mice via MRI.	[112]
SPION	Mouse macrophages	Used to image (MRI) target drug delivery by exosomes for glioma in mouse models.	[113]
FTH1	Viral transfected MSC	Indirect labeling of exosomes detectable in mice via MRI.	[118]
Gaussia luciferase–lactadherin	Viral transfected Murine melanoma cells	In vivo detection of exosome in mice via fluorescent imaging	[119]
^99^Tc-tricarbonyl	Erythrocytes	Used to image the biodistribution of EVs in mice.	[121]
^99^Tc-HMPAO	Exosome mimicking nanovesicles from mouse macrophage cells	For quantitative measurement of in vivo clearance of exosome mimicking nanovesicles from mice with the help of CT scanning.	[120]
^111^In	Melanoma cells	Used to image the biodistribution of EVs in mice.	[124]

[Abbreviations—MSCs, mesenchymal stem cells; EVs, extracellular vesicles; MRI, magnetic resonance imaging; USPION, ultra-small superparamagnetic iron oxide nanoparticles; SPION, superparamagnetic iron oxide nanoparticles; FTH1, ferritin modified with heavy chain protein; HMPAO, Hexamethylpropyleneamineoxime; CT, computed tomography].

## 7. Preclinical Studies of EV Treatments Applied to Cerebral Ischemia

Experimental studies provide compelling evidence that extracellular vesicles (EVs) play important therapeutic roles in targeted cargo delivery to the brain [125]. Their nanoscale size, lipid bilayer structure, and biocompatibility allow them to cross the blood–brain barrier (BBB) without eliciting an immune response, making them ideal candidates for drug delivery [126]. Moreover, EVs maintain membrane integrity and cargo stability in circulation and storage, which is crucial for clinical scalability. Functionally, EVs have been shown to modulate nerve regeneration, synaptic plasticity, immune responses, and intercellular communication, all of which are vital for post-stroke neural repair. They potentially can target nerve regeneration, synaptic function, plasticity, immune response, and EV-mediated intercellular communication, contributing to neural regeneration [127,128]. Across a range of pathologies, the initial justification for the use of certain EVs is predicated on the known or potential benefits of the cell source against ischemic stroke [128,129]. Most research for therapeutic effects on ischemic stroke uses EVs derived from stem cells or native brain cells.

### 7.1. Evident Preclinical Studies of EV Treatments—The Motivation for Stem Cell-Derived EVs

EVs as a therapeutic agent are driven by the effects of stem cell therapy itself and the likely mode of action [130]. For example, in stroke treatment, mesenchymal stem cells (MSCs) impact the production of new neurons and increase angiogenesis [64,131,132]. MSCs are multipotent, self-renewing exogenous cell populations present in adults, as well as developing individuals, and may differentiate into neuron-like and glial-like cells [132,133]. There is significant research on primary MSCs isolated from umbilical cord blood, umbilical cord tissue, bone marrow, adipose tissue, hematopoietic-supporting stroma, and other organs using explant culture and enzymatic digestive methods (Table 3) [132]. Some MSCs are also derived from iPSCs. Most studies prefer bone marrow-derived MSCs for their angiogenic properties and ease of culture [132,134]. MSCs have been found to act on patients’ cells to repair diseased tissues [133]. The beneficial proteins and other therapeutic cargo involved in MSC therapy are also present in secreted EVs. Via proteomics and genetic analysis, more than 850 unique gene products and 150 miRNAs have been found in MSC-derived EVs [134,135]. In case of stem cell therapy, MSCs can face interference in vivo before reaching the brain by the kidney and liver in systemic administration. Even MSCs transplanted in a stroke-induced rodent model have a high clearance rate via the kidney and liver [136].

Many researchers have demonstrated that the primary therapeutic effects of mesenchymal stem cells (MSCs) are mediated through paracrine signaling, with extracellular vesicles (EVs) playing a central role in this mechanism [137]. The composition of MSC-derived EVs is highly sensitive to culture conditions, particularly oxygen tension and media formulation. Notably, EVs derived from hypoxia-preconditioned MSCs exhibit superior therapeutic efficacy in ischemic stroke models compared to those from normoxic cultures [138,139,140]. This is likely due to the upregulation of pro-survival and angiogenic factors under hypoxic stress, which are subsequently packaged into EVs.

Systemically administered EV proteins from MSCs in male rats, stroked through middle cerebral artery occlusion (MCAO), induced neurological recovery by a combination of different mechanisms involving long-term neuroprotection, promotion of neurogenesis, and angiogenesis in the penumbra region [141]. A comparative study evaluated EVs from adipose-derived MSCs (ASCs) and bone marrow-derived MSCs in a model of transient global cerebral ischemia [142]. Using behavioral assays (e.g., Morris water maze) and molecular markers (e.g., COX-2 expression), the study found that BM-MSC-EVs significantly improved cognitive function and suppressed inflammatory markers, whereas AD-MSC-EVs reduced neuronal death but did not affect lesion size. This suggests that while both EV types confer neuroprotection, BM-MSC-EVs may offer broader therapeutic benefits, possibly due to their richer cargo of angiogenic and anti-inflammatory molecules. However, the use of intracerebroventricular injection in the study, while effective, limits clinical translatability due to its invasiveness [143].

Beyond MSCs, induced pluripotent stem cells (iPSCs) and neural stem cells (NSCs) have also been explored as EV sources. EVs from iPSC-derived MSCs (iMSCs) have shown promising results in MCAO models, including reduced lesion volume, attenuated neurodegeneration, and enhanced angiogenesis and axonal plasticity [144]. Although iPSC-EVs have been implicated in both pathogenic and therapeutic roles in neurological diseases, their application in stroke remains underexplored in vivo [145].

In large animal models, NSC-derived EVs have demonstrated robust neuroprotective effects. In a porcine MCAO model, human NSC-EVs reduced lesion volume, brain swelling, and midline shift, and improved functional recovery when administered intravenously within 2–24 h post-stroke [146,147]. These findings are particularly valuable given the anatomical and physiological similarities between pigs and humans. Moreover, a murine study comparing iMSC- and NSC-derived EVs to MSC-EVs found that the former two provided superior functional recovery, likely due to enhanced immunomodulatory effects [148]. In that study, the results showed that NSC-derived EVs show better functional recovery than MSC-derived EVs through controlling immune response in the stroke area [148]. Preconditioning of NSCs might be beneficial for future application of EV therapy, as it improved the survival of NSCs in vivo [149].

**Table 3:** EV therapeutics for clinical and preclinical studies.

EV Source	Stroke Model, Administration Route, and Observation Period	Outcome	Reference
Rat bone marrow-derived MSCs	Male Wister rat MCAO model, Tail vein injection, 28 days	Reduction in stroke lesion volume and better functional recovery were reported. Long-term neuroprotection, promotion of neurogenesis, and angiogenesis also were observed in histopathology.	[141]
Mouse bone marrow-derived MSCs	Male C57BL/6J mice, transient global cerebral ischemia model, intracerebroventricular injection, 7 days	Significant reduction in stroke lesion volume and functional recovery. Lesion site restored partially as well as hippocampal and basal synaptic plasticity improved.	[142]
Mouse adipose-derived MSCs	Male C57BL/6J mice, transient global cerebral ischemia model, intracerebroventricular injection, 7 days	Not significant recovery was noticed compared to placebo mice.	[143]
Mouse adipose-derived MSCs	Male C57BL/6 mice MCAO model, right femoral vein injection, 14 days	A noticeable reduction in stroke lesion volume was not observed. But better functional recovery and less neural death in the penumbra region was observed in immunohistochemistry.	[144]
Human iPSC-derived MSCs	Male C57BL/6J mice MCAO model, intravenous injection, 28 days	Significant reduction in stroke lesion volume and better functional recovery. Promotion of angiogenesis and axonal plasticity were also observed.	[145]
Human NSCs	Porcine MCAO model, intravenous injection, 84 days	Significant reduction in stroke lesion volume and edema and improved functional recovery.	[146]
Human NSCs	Porcine MCAO model, intravenous injection, 84 days	From MRI, midline shift recovery was considered a recovery metric. It improved in treated animals along with anti-immune response and better functional recovery.	[147]
Human MSC and NSC	Murine thromboembolic stroke model, tail vein injection, 24 days	NSC-derived EVs had a more positive outcome than MSC-derived EVs. NSC-derived EV had better anti-immune factors that resulted in reduced lesion size and better functional recovery.	[148]

[Abbreviations—MSCs, mesenchymal stem cells; EVs, extracellular vesicles; MRI, magnetic resonance imaging; MCAO, middle cerebral artery occlusion; iPSCs, induced pluripotent stem cells].

### 7.2. Challenges in Bridging Preclinical Findings to Clinical Application

While EVs from various stem cell sources show overlapping benefits, BM-MSCs remain the preferred source due to their ethical acceptability, ease of expansion, and consistent therapeutic output. EVs from native brain cells like astrocytes have shown neuroprotective effects in vitro [150,151], but they lack in vivo validation, highlighting a gap in translational research.

Efforts to engineer EVs for targeted drug delivery are also gaining traction. Modified EVs have successfully delivered genetic cargo to the brain in murine models [152]. Until now, EVs have been modified successfully to target the brain to deliver genetic cargo in mice [153,154], offering a platform for enhancing neural plasticity and regeneration. However, these approaches require rigorous validation to ensure safety, targeting specificity, and reproducibility.

Despite encouraging preclinical data, translational challenges persist. Most stroke models rely on rodents or pigs, which only partially replicate human stroke pathology. For instance, while MCAO mimics large artery occlusion—a subtype accounting for 24–46% of ischemic strokes (in the human patient population, 87% of all strokes are ischemic strokes)—it does not represent the broader spectrum of ischemic events, such as transient ischemic attacks (TIAs) [155]. TIAs recur in 17% of human patients within 6 h, which is not usually replicated in animal models [156]. Models like photothrombotic stroke, though convenient, lack clinical relevance as they induce superficial cortical lesions not typically seen in human stroke. In the best-case scenario, mostly mechanical and some degree of cellular aspects of the disease are mimicked in animal models [157]. There is also no way to evaluate psychological effects in preclinical studies [158]. Moreover, stroke recurrence, a common clinical feature, is rarely modeled in animals. So, it is necessary to find a suitable animal stroke model to translate into clinical applications.

The route of administration significantly influences EV biodistribution and therapeutic efficacy (Table 4). While oral delivery is not feasible, intravenous (IV) injection is the most clinically relevant and commonly used route in preclinical studies. However, IV delivery suffers from rapid clearance by the liver, spleen, and kidneys and pulmonary entrapment, which limits brain accumulation [159,160]. Though it is the most clinically relevant administration route, its rapid clearance and low homing capabilities make the effective dose way higher [160,161]. Intra-arterial (IA) injections, particularly via the internal carotid artery, offer more direct brain access but are more invasive and technically demanding. Intranasal delivery provides a non-invasive alternative with limited but detectable brain uptake, though its clinical scalability needs further investigation [162]. Some administration routes, such as peritoneal (through the stomach), intra-muscular (through the muscles), and subcutaneous (under the skin through fat cells) administration routes, are not the ideal route of administration for stroke treatment due to homing capabilities. The route impacts the required initial dose and eventual clearance of EVs, reflecting the tortuous path that a systemic delivery must follow to reach the brain [160].

Intracerebral and intrathecal routes offer the most direct CNS delivery. Intracerebral injection ensures high local concentrations but is highly invasive and infection-prone, limiting its clinical utility [163]. Intrathecal administration, while still invasive, allows broader CNS distribution via cerebrospinal fluid and is more clinically scalable [164]. However, both routes require further validation in human studies.

Finally, EV clearance kinetics are influenced by particle size, cell source, and animal model. Even among closely related species, EV uptake and retention vary, and brain residence time remains short, posing challenges for sustained therapeutic effects [165].

**Table 4:** Routes of administration in preclinical models for EVs with their benefits and drawbacks.

Route	Description	Benefits	Drawbacks	Reference
Intravenous (IV)	Systemic injection into the bloodstream	- Minimally invasive. - Clinically relevant. - Allows repeated dosing.	- Limited brain targeting due to the blood–brain barrier (BBB). - Rapid clearance by RES organs. - Requires a higher dose. - Poor reproducibility. - Limited EV uptake evidence.	[159,160]
Intranasal	Delivery through the nasal cavity	- Bypasses the BBB. - Non-invasive. - Direct access to the brain via olfactory/trigeminal pathways. - Retains honing potential. - Lower dose of EVs required.	- Variable absorption. - Limited dosing volume. - Less standardized in clinical settings.	[164]
Intracerebral	Direct injection into the brain parenchyma	- High local concentration. - Precise targeting of the ischemic region. - Avoids systemic circulation.	- Highly invasive (performed via skull penetration) - Risk of tissue damage and infection. - Poor clinical translatability.	[163]
Intrathecal	Injection into cerebrospinal fluid (CSF) via lumbar puncture	- Bypasses the BBB. - Direct CNS exposure. - Potential for broader brain distribution.	- Invasive procedure. - Requires technical expertise. - Risk of infection.	[164]
Intra-arterial (IA)	Injection into cerebral arteries (e.g., internal carotid)	- Enhanced delivery to the ischemic brain. - Potential for targeted perfusion.	- Risk of embolism or vascular injury. - Technically demanding. - Limited repeatability. - Requires a higher dose.	[162]
Intraperitoneal	Injection into the abdominal cavity or gastrointestinal (GI) tract	- Common in rodent models. - Easy to perform.	- Poor CNS penetration. - Limited relevance to stroke therapy in humans.	[159,161]
Subcutaneous	Injection into the fat layer under the skin	- Easy and minimally invasive. - Sustained release. - Clinically relevant.	- Systemic delivery. - Limited brain uptake evidence. - Not typical for stroke treatment.	[161]

## 8. Challenges in the EV Production Process

EVs can be used as a biomarker to detect the progression of stroke. They can also be used as a therapeutic treatment for the rehabilitation of stroke or as a drug delivery method. Even with this potential, there exist challenges to the institution of EV therapy as a viable alternative for stroke treatment. The challenges are related to the mechanistic understanding of the bioengineering of EVs, the standardization and manufacturing protocol setup, the targeting and delivery of EVs in patients, difficulties related to clinical translation, and, finally, comparative studies.

The first challenge is controlling the EV biogenesis process with respect to EV yield as well as the encapsulated cargo. Most of the factors controlling this process are not well known; however, a few factors have been identified that impact biogenesis. Such factors are different membrane tetraspanins (such as CD63 and Tspan8), which can affect the protein sorting process of EVs [166,167]. Proteins like ESCRT are also important for sorting and transporting proteins to EVs, but not all varieties of ESCRT structures have been discovered. If the membrane of EVs can be modified or bioengineered, it can also improve target specificity. To control the maximum yield of EVs from stem cells, the biogenesis mechanism and the molecules controlling the mechanism must be brought to light.

A second challenge is the more direct insertion of materials (either pharmaceutical or labeling) into the EV itself. Source cells can be incubated with small-sized compatible drugs to incorporate pharmaceuticals as cargo [168]. But the method has low loading capacities and depends on the condition of donor cells. The process is similar to indirect labeling. EVs can also be loaded after purification. Purified EVs can be incubated with hydrophobic drugs or proteins, which attach to their membranes [169]. EVs can also be loaded with the help of sonication [170]. Sonication makes the EV membrane transiently permeable to permit drug uptake or labeling. EVs also can be loaded by permeabilization with saponin, electroporation, freeze–thaw cycles, and extrusion. Saponin is used to chemically permeate the EV membrane, but saponins are cytotoxic and hard to eliminate [171,172]. Electroporation increases membrane permeability by applying electric forces [171]. The extrusion process uses lipid extruders to disrupt the EV membrane mechanically to load the cargo. It is more effective than other methods [173]. But these methods can affect the membrane of EVs negatively. Freeze–thaw cycles can help to load cargoes in EVs using milder conditions. Though the method is relatively non-aggressive to the membrane structure and bioactive cargo, it is a time-consuming process [174].

A third challenge is the optimal preconditioning of source cells in culture to achieve favorable EV products. Cell culture conditions affect the cargo of EVs. Compared to normoxic culture (atmospheric oxygen concentration) conditions, hypoxic culture (e.g., 2% oxygen concentration) makes cells more robust and suitable for therapeutic applications in ischemic stroke [175]. Several researchers have demonstrated that EVs derived from stem cells cultured in hypoxia have better therapeutic outcomes for ischemia [176,177]. But hypoxia may not be conducive to cell expansion [178], which would limit EV yield under hypoxic environments. As for the quantity and quality of EVs, cells cultured in bioreactors or 3D culture conditions yield more EVs compared to 2D culture [179]. In 3D culture, it can be scaffold-free or with scaffolds. In most tissue cultures, scaffolded 3D culture is used. Scaffold-free 3D culture uses single-cell suspension, spheroids or aggregates, tissue strands, or cell sheets. To produce EVs from stem cells, vertical wheel bioreactors (VWBs) with cell suspension or with micro-carriers have gained attention recently. VWBs can reduce the sheer force more than any other single suspension bioreactors. This has not only increased stem cell production but also made the operation scalable to achieve therapeutic yields of EVs [41,44,180].

For biomarker purposes, the most pressing issue in collecting EVs (collected from biofluids) is low yield. If the biomarkers are genetic material, then for proper analysis, a little biofluid might not produce any result because, usually, one miRNA is found per 100 EVs [181]. Furthermore, in the EV production process, the purification steps are complicated and expensive. Most commonly, it requires xeno-free or EV-depleted cell culture medium and multiple stages of ultra-centrifugation [54]. The homogeneity in purified EVs is also a matter of concern. EVs collected from different donors of the same cell type or different passages of the same donor do not provide uniform cargo due to the heterogeneity in parent cells [182].

Finally, regulatory and ethical considerations are essential for clinical implementation. Agencies like the FDA and EMA have yet to establish comprehensive guidelines for EV-based therapeutics. Key concerns include potency assays, donor screening, manufacturing consistency, and long-term safety. Ethical issues—such as donor consent, biobanking, and commercialization—must also be addressed to ensure responsible development and public trust. A lack of guidelines has limited clinical translation, though several trials are underway. The EXO4STROKE trial (NCT05370105) is investigating EVs as biomarkers for stroke recovery, while other studies are exploring scalable EV production and delivery strategies. These efforts highlight the need for standardized characterization, validated dosing, and reproducible delivery routes.

## 9. Conclusions

Ischemic stroke has a lasting impact and remains a leading cause of disability. Though promising for the rehabilitation of ischemic stroke, EV research is still in a nascent phase, whether in use as a diagnostic/prognostic tool or therapeutic. Diagnostically, endogenous EVs in blood serum or directly secreted by neural cells may provide novel biomarkers for ischemia, assessing the severity, recovery potential, and stage of stroke. Therapeutically, EVs from exogenous sources offer a delivery system to cross the BBB. There exist several barriers to translating EV therapy to clinical trials. The biogenesis mechanisms of EVs will need to be more fully understood in order to upscale production in an optimal process that yields the most therapeutic cargo. To achieve this goal, the most beneficial EV components (miRNAs, proteins, etc.) and their modes of action must be defined for ischemic stroke; then, cell culture systems must be optimized to generate these cargoes efficiently. For now, MSCs have produced the most therapeutic EVs. But co-culturing with other stem cells may increase therapeutic effects and may increase target specificity. Also, culturing cells under a low-shear stress in a 3D bioreactor system can increase the yield of production. After ensuring sufficient production, EV therapies have to be evaluated thoroughly in preclinical models to justify EV safety and efficacy prior to clinical trials.

Despite the challenges, current EV therapeutics research at the preclinical level has been applied to many disease models, including cancer and stroke. Unlike tumor treatments, where EVs from tumor cells are used to deliver therapeutic agents, a cell source (e.g., bone marrow, adipose, Wharton’s jelly, or cord blood umbilical cells) is typically used to generate EVs that will be delivered to a completely different cell target, namely, ischemic neural cells. As such, this functionality is beyond the normal cell-to-cell communication of EVs, and targeted delivery becomes less directed. Additional research should evaluate EV biogenesis mechanisms so that they can be bioengineered to enhance target specificity while optimizing effective cargo loading and enabling mass-scale production. Overcoming this challenge is necessary to realize a successful EV therapy for clinical stroke recovery.

## Data Availability

No new data were created or analyzed in this study.

## Glossary

EVs	extracellular vesicles
MSCs	mesenchymal stem cells
NPCs	neural progenitor cells
miRNA	micro-ribonucleic acid
mRNA	messenger ribonucleic acid
lncRNA	long non-coding ribonucleic acid
circRNA	circular ribonucleic acid
siRNA	small interfering ribonucleic acid
ISEV	International Society for Extracellular Vesicles
MVBs	multivesicular bodies
BBB	blood–brain barrier
ESCRT	the endosomal sorting complex required for transport
ASCs	adipose tissue-derived mesenchymal stem cells
MRI	magnetic resonance imaging
MCAO	middle cerebral artery occlusion
iPSC	induced pluripotent stem cells
CT	computed tomography
PET	positron emission tomography
CircOGDH	oxoglutarate dehydrogenase
CSF	cerebrospinal Fluid
EM	electron microscopy
NTA	nanoparticle tracking analysis
DLS	dynamic light scattering
SPION	superparamagnetic iron oxide nanoparticles
USPION	ultra-small superparamagnetic iron oxide nanoparticles
